# Automatic PCB Sample Generation and Defect Detection Based on ControlNet and Swin Transformer

**DOI:** 10.3390/s24113473

**Published:** 2024-05-28

**Authors:** Yulong Liu, Hao Wu, Youzhi Xu, Xiaoming Liu, Xiujuan Yu

**Affiliations:** School of Mechanical Engineering, Anhui University of Technology, Maanshan 243032, China; dulyam@163.com (Y.L.); huoxula@163.com (Y.X.); lxm333@ahut.edu.cn (X.L.)

**Keywords:** ControlNet, transformer, stable diffusion model, PCB, defect detection

## Abstract

In order to improve the efficiency and accuracy of multitarget detection of soldering defects on surface-mounted components in Printed Circuit Board (PCB) fabrication, we propose a sample generation method using Stable Diffusion Model and ControlNet, as well as a defect detection method based on the Swin Transformer. The method consists of two stages: First, high-definition original images collected in industrial production and the corresponding prompts are input to Stable Diffusion Model and ControlNet for automatic generation of nonindependent samples. Subsequently, we integrate Swin Transformer as the backbone into the Cascade Mask R-CNN to improve the quality of defect features extracted from the samples for accurate detection box localization and segmentation. Instead of segmenting individual components on the PCB, the method inspects all components in the field of view simultaneously over a larger area. The experimental results demonstrate the effectiveness of our method in scaling up nonindependent sample datasets, thereby enabling the generation of high-quality datasets. The method accurately recognizes targets and detects defect types when performing multitarget inspection on printed circuit boards. The analysis against other models shows that our improved defect detection and segmentation method improves the Average Recall (AR) by 2.8% and the mean Average Precision (mAP) by 1.9%.

## 1. Introduction

**Figure 1:**
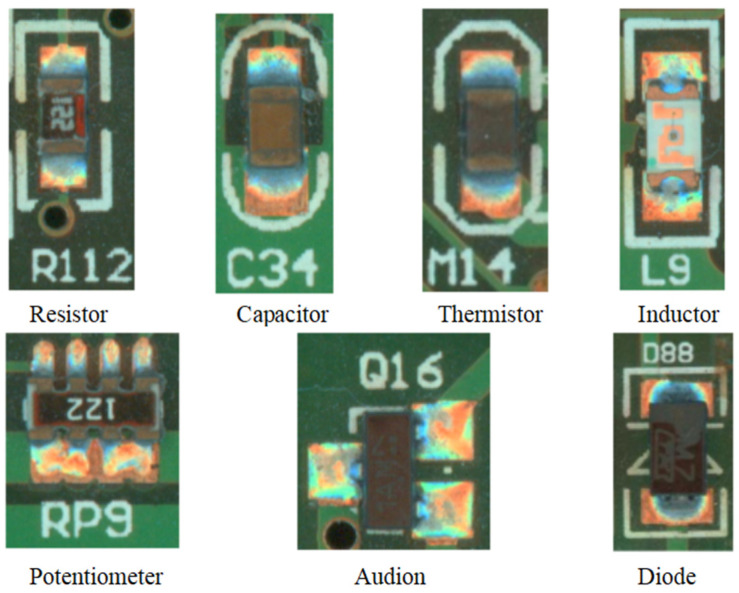
Typical surface mount components.

The Surface-Mounted Technique (SMT) is a method of soldering surface-mounted components such as resistors, capacitors, inductors, and diodes onto Printed Circuit Boards through reflow soldering [1]. Due to the unpredictability of the reflow soldering process, solder paste may randomly produce soldering defects that affect the performance of the PCB after heating and cooling [1,2]. Current soldering defect detection methods involve segmenting and extracting specific components on the PCB for defect detection. However, these methods require high-precision segmentation and the extraction of densely packed components, thus greatly reducing the efficiency of the entire PCB inspection process. In this study, we propose a new method that does not require the segmentation of individual components. Instead, it performs sample recognition and defect detection on all surface-mounted components within a larger area of view, thereby improving the efficiency of PCB inspection. The target of this study is illustrated in Figure 1.

It is difficult to collect enough samples to produce a dataset in industrial production. Previous methods extracting independent targets for detection through segmentation were able to empirically categorize the samples into defective and nondefective types, and then the samples were massively expanded using Generative Adversarial Networks (GANs) [3,4]. However, when a sample image contains both defective and nondefective targets, the adversarial generative network cannot be trained effectively. The Stable Diffusion Model [5] provides a useful method by feeding an arbitrary image into ControlNet [6] for feature extraction and controlling the output of the diffusion model, which is then fed into the Stable Diffusion Model along with appropriate prompts. By extracting the image features and adding noise, combined with the features of the prompts, a forward diffusion process and a reverse diffusion process can be used to obtain an image with pixel-level differences from the original image. In addition, when the image generated by this method is used to produce a standard dataset, the labels used are identical to those of the original image, so the image data expanded by this method can be used for model training without further labeling. With this method, the original dataset can be quickly expanded to a level sufficient to train a target detection model.

Widely used target detection networks such as Faster-RCNN [7], Mask-RCNN [8] and Cascade R-CNN [9] have been extensively used in the field of industrial defect detection and have achieved satisfactory results [10,11]. However, when confronted with multitarget detection of dense surface-mounted components, the above target measurement algorithms cannot realize high-precision detection due to the small differences between different targets and the lack of strict boundaries between the defect features and the background [12]. We adopt Swin Transformer [13] as the backbone network for feature extraction from images. Based on the self-attention algorithm [14], the Swin Transformer architecture is able to obtain the feature information of the object in different dimensions, effectively analyze targets of different sizes, and highlight the differences between the object and the background to achieve high-precision detection. At the low dimension, it is able to capture the subtle feature information of the target, and by merging the windows to obtain the high-dimensional feature information, it obtains the long-range dependence on the pixel features, thus focusing on the global features of the image. 

The multidimensional features are input to a multiscale feature fusion network (or Feature Pyramid Network, FPN) [15] for feature fusion to obtain a fixed-size feature image, and then the Cascade Mask R-CNN is used as the head network to perform regression computation of the detection frames for the feature image and to classify and segment the feature image; then, the tandem detection box localization structure is used to classify the image by different threshold sizes, and the final result is inferred based on the classification results [9]. The defect detection experiments on multiple targets in multiple field of view regions show that the improved Cascade Mask R-CNN detection algorithm using Swin Transformer can achieve accurate classification and localization segmentation for all targets within the field of view.

The contribution of this study is three-fold:

- Using ControlNet and Stable Diffusion for the automatic generation of nonindependent samples to achieve fast and automatic expansion of the dataset, thus validating the effectiveness of the method;
- Using Swin Transformer as the backbone, combined with the Cascade Mask R-CNN, to obtain a highly accurate multiscale object detection model;
- The proposed detection method is able to simultaneously detect defects on multiple surface mount components in the field of view in a larger area, which improves the overall PCB inspection efficiency.

The article is organized as follows. In Section 2, we analyze the development process of diffusion modeling and the research progress in the detection of soldering defects in surface-mounted components on PCBs, as well as the application of the latest research methods in the field of target detection. Section 3 gives a theoretical introduction to our proposed method, thus describing the specific process of expanding the dataset using ControlNet and Stable Diffusion, and the way of combining Swin Transformer and the Cascade Mask R-CNN. The experimental data and results are presented in Section 4, and comparisons are made with previous methods. The work of this paper is summarized in Section 5.

## 2. Related Work

The occurrence of defects in the production process of electronic components constitutes a small probability, and obtaining enough defect samples requires a great deal of work. However, the training of neural networks requires large and balanced data samples. In previous studies, researchers have been accustomed to using adversarial generative networks [4] to acquire images and their corresponding masks in large quantities. Liu et al. [16] used CycleGAN [3] to generate defective samples for Liquid Crystal Display (LCD) to realize the expansion of the sample dataset. Tsai et al. [17] used CycleGAN to acquire defective samples while obtaining their corresponding masks to improve the efficiency of the dataset production. However, since adversarial generative networks need to strictly categorize samples into two mutually different classes for adversarial training, this type of method is only applicable to datasets that can be distinguished into defective and nonindependent samples. When the images in the samples contain both defective and normal targets, which form the nonindependent sample dataset, a suitable GAN model cannot be successfully trained.

Since Denoising Diffusion Probabilistic Models (DDPMs) [18] were proposed, the Diffusion Model has been widely used in the field of image generation and beat GAN model [19] in terms of generation quality and other aspects. Denoising Diffusion Implicit Models (DDIMs) [20] improve the noise level during the reverse diffusion process in the Diffusion Model, as well as optimize the recursive formula and achieve better results on fewer inference steps. With the success of research in the field of natural language processing such as Variational Auto-Encoders (VAE) [21] and Contrastive Language–Image Pre-training (CLIP) [22], GLIDE [23] based on the Diffusion Model, DALL-E2 [24], Imagen [25] and other Text-to-Image models have been successively proposed to achieve high-definition images that match the description by inputting only textual information.

Saharia et al. [26] proposed Image-to-Image model for the Palette Model, which enabled the Diffusion Model to achieve tasks such as image coloring, image restoration, and image super-resolution reconstruction, thus showing people the powerful potential of Image-to-Image technology. After that, Zhao et al. proposed the DDFM [27] for the first time to input multimodal information to the Diffusion Model. Rombach et al. [5] proposed the Latent Diffiusion Model, which performs encoder–decoder operations on the latent domain and improves the high-resolution image training and generation efficiency. As the generation quality of the Diffusion Model increases, the corresponding model magnitude becomes increasingly large. Zhang et al. [6] proposed ControlNet to improve the control of the input and output of the Diffusion Model, which made the application of large model specificity possible. Since Image-to-Image generation with multimodal inputs in the Diffusion Model does not rely on the association between images and only requires the provision of the original image with certain features and appropriate prompts to obtain a high-quality image that is similar to the original image but with slight gaps, a large expansion of the dataset of nonindependent samples can be realized using this method.

Deep learning-based methods learn the feature information of images by training neural networks to achieve the automated recognition of complex multiclass inspection objects. Soukup et al. [28] used the CNN to analyze images captured by stereo light to achieve the detection of surface defects on rail steel. Xu et al. [29] used the Mask R-CNN to detect surface defects on rails and debris between rails. Wu et al. [30] used the Mask R-CNN to detect soldering defects on independent surface mounted components on PCBs. Solorzano et al. [31] used the cosine convolutional as a backbone to improve the Mask R-CNN model to achieve the detection of surface defects on semiconductor devices. Due to the presence of a large number of surface-mounted components to be detected in the PCB sample image, the above methods cannot achieve satisfactory results in solder defect detection when facing multiple randomly distributed targets.

Self-attention mechanisms using a unique Transformer architecture are able to automatically focus on the region of interest and extract the multidimensional features of the target during the processing of complex images, which improves the efficiency of recognizing and extracting the features of the target relative to the previous convolutional neural network. Fan et al. [32] combined Swin Transformer with U-Net [33] to realize noise reduction and the high-resolution reconstruction processing of images. Chen et al. [34] combined Swin Transformer and YOLOv5 network to achieve the effective detection of line defects on PCBs by optimizing the acquisition of feature images. Liu et al. [12] used Swin Transformer as a backbone to optimize the Mask R-CNN to achieve the successful detection of pin soldering defects on integrated circuits. However, the structure of the Mask R-CNN as the head network is not able to achieve high accuracy detection for objects of multiple sizes simultaneously. The Cascade head network proposed in this paper is able to use different detection thresholds to handle different objects. The above studies have collectively verified the excellent performance of Swin Transformer architecture in feature extraction, which is able to better learn the feature information of an image.

## 3. Proposed Method

### 3.1. Automatic Sample Generation Based on ControlNet and Stable Diffusion Model

Since PCB sample images contain multiple surface mount components at the same time, the collection of PCB samples containing defects is more difficult, and the high-quality Img2Img feature of the Stable Diffusion Model provides a solution for the automatic generation of PCB samples. In a pre-trained Stable Diffusion Model, when an image is input, the input sample image is encoded using the VAE [21] to obtain the Latent features of the input image, and then the image is sampled by the DDIM sampler [20], and noise injection is performed. At the same time, the multimodal information input as text is converted into prompts and encoded by CLIP [22], and the obtained text features, fused with the features of the image in the implicit space, increase the control of the image output from the model. After completing the noise diffusion and multimodal feature fusion of the samples, the Latent features added by the noise are sampled with the same degree of sampling and decoded using the VAE; a high-quality image similar to the original image can be obtained, and the purpose of the data augmentation can be realized. The image generated in this way contains feature information that is compatible with the original image and the prompts, but there are large differences in the spatial structure and logical distribution. Therefore, when the Stable Diffusion Model is directly used to obtain PCB samples, the images obtained are too different from the original image, and they even do not conform to the objective facts.

The addition of ControlNet [6] enables us to further control the Stable Diffusion Model. Before the image is input into the model, ControlNet is used to extract features from the image, such as extracting the Canny features or Depth features of the image, and the extracted feature images are simultaneously input into the Stable Diffusion Model to achieve further control of the model output. These features are combined with the target text information, and through the iterative process of the diffusion model, images similar to the input sample images in terms of spatial location and logical distribution are gradually generated. Meanwhile, we found that the features required to be labeled empirically are the same as the original image when the generated image is labeled using LabelMe [35] at a lower redraw intensity.

**Figure 2:**
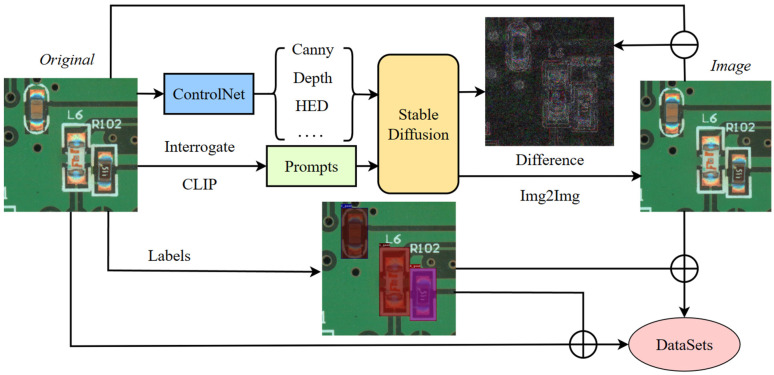
Automatic sample generation method based on ControlNet and Stable Diffusion Model.

In the sample generation process of the PCB, as shown in Figure 2, the spatial features of the original image are extracted using ControlNet as the control input of the image, while the prompts of the sample are deduced using inverse CLIP. The original image and the corresponding prompts are inputted into the Stable Diffusion Model, and a high-quality image can be obtained by going through a forward diffusion process and a reverse diffusion process. Combining this image with the labels of the original image can realize the fast and automatic generation of the dataset.

### 3.2. Object Detection and Segmentation Based on Cascade Mask R-CNN

U-Net [33] is a classical convolutional neural network structure with a symmetric encoder–decoder structure that achieves information transfer from the global context to the local details through downsampling and upsampling paths. Faster R-CNN [7] introduces a candidate Region Proposal Network (RPN) and a region classification network to achieve fast and accurate object detection, thus optimizing the bounding box localization bias due to the computational method. Mask R-CNN [8] introduces an additional branching network, which enables the model to simultaneously generate pixel-level masks for each detected object, thus simultaneously realizing both object detection and semantic segmentation and improving the visual effect of target detection. ROI Pooling [7] and ROI Align [8] are two commonly used region of interest (ROI) pooling methods for extracting features inside ROIs from feature maps. ROI Pooling achieves feature extraction by dividing the ROI into fixed-size grids and pooling the maximum value of each grid, but this method suffers from information loss. In contrast, ROI Align accurately extracts features by performing bilinear interpolation on the ROIs, thus avoiding information loss and improving localization accuracy.

Cascade Mask R-CNN further improves on Mask R-CNN by cascading multiple perceptrons to gradually filter out false positives, reduce mismatch, and improve detection performance and accuracy. The cascade structure consists of multiple stages; the output of the previous stage is used as the input of the subsequent stage, and each stage adopts an incremental object detection threshold for filtering out the candidate frames that are considered as positive samples in that stage. Through the progressive filtering with different thresholds, the false detection rate and the leakage rate can be greatly reduced, so the cascade structure can more reliably deal with the multitarget detection tasks in various complex scenarios.

The above-mentioned object detection networks all use ResNet [36] or VGG [37] as the backbone network, and these convolutional neural networks use a fixed-size convolutional kernel and pooling layer, which tends to ignore long-distance feature information when sampling the samples. And with the deepening of the convolutional layer, the contour information of the object will be lost rapidly, so when facing the detection of soldering defects of the surface-mounted components on a PCB, it is not possible to detect and recognize the solder joints and different component appearances at the same time in a subtle way.

### 3.3. Swin Transformer Backbone

**Figure 3:**
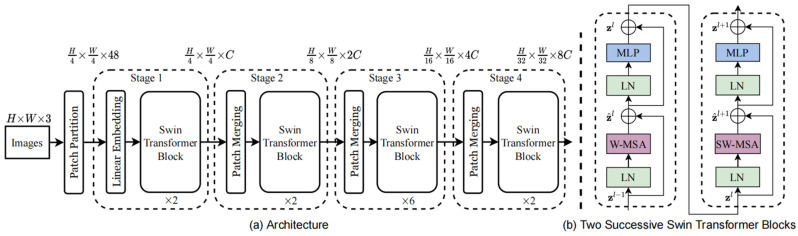
(**a**) Swin Transformer backbone. (**b**) Two-stage Swin Transformer Block [13].

Swin Transformer serves as a generalized visual detection backbone network with a unique layered transformer architecture that enables attention computation based on nonoverlapping windows for a better focus on global features and maintains a linearly increasing computational complexity based on the number of windows [13]. The network structure is shown in Figure 3a. The Swin Transformer architecture consists of a base feature extractor and a multilayer encoder. The feature extractor splits the input image into nonoverlapping chunks and then inputs them into the layered Swin Transformer Block (ST Block, shown in Figure 3b) for obtaining semantic information at different scales.

At an ST Block, there exists a multihead self-attention layer (W-MSA) and a shift window-based multihead self-attention layer (SW-MSA). After each of these attention layers, there is a Multilayer Perception (MLP) based on the Gaussian Error Linear Unit (GELU) function. A residual connection is established between each of the two attention layers, which is used to avoid the training gradient problem and the loss of the features, and a one-time Layer Normalization (LN) is used before each computation.

(1) $${\hat{z}}^{l}=\mathrm{W-MSA}(LN(z^{l-1}))+z^{l-1}$$

(2) $$z^{l}=MLP(LN({\hat{z}}^{l}))+{\hat{z}}^{l}$$

(3) $${\hat{z}}^{l+1}=\mathrm{SW-MSA}(LN(z^{l}))+z^{l}$$

(4) $${\hat{z}}^{l+1}=\mathrm{SW-MSA}(LN(z^{l}))+z^{l}$$

(5) $$Attention(Q,K,V)=SoftMax(QK^{T}/\sqrt{d}+B)V$$

The Equations (1)–(4) describe the feature exchange relationship between the MSA and the MLP. *l* is a particular self-attention layer, $\hat{z}$ and *z* denote the feature information output by MSA and MLP.

In an MSA, the attention is computed between different encoding windows, each window is defined as a Key matrix K, and a corresponding Value matrix V is built for each Key to store the value of attention; finally, a Query matrix Q is built for Query to query and compute the attention for each set of K and V. Equation (5) shows the scaled dot product model for computing attention, where *d* is the dimension of Q and K, while B is the relative position bias.

### 3.4. Defect Detection Method on Swin Transformer-Based Cascade Mask R-CNN

In our proposed method, Swin Transformer was used as a backbone combined with the Cascade Mask R-CNN to improve the performance of object detection and semantic segmentation tasks further. By introducing the Swin Transformer architecture, which relies on its unique multihead self-attention mechanism and multiscale window segmentation mechanism to optimize the feature extraction of the input image, we are able to better capture the multiscale information in the image, thus improving the model’s detection and segmentation accuracy in the complex background of the PCB, thus using Swin Transformer as the backbone network instead of ResNet.

The multiscale feature fusion network (Feature Pyramid Network, FPN) [15] was used as a neck for the fusion of different scales of feature images output from four stages to obtain the feature map. Then the Region Proposal Network (RPN) [8] was used for the generation and localization of the detection boxes to obtain the proposals in the feature map. The proposed feature regions were input into the Cascade Head, and the candidate boxes were filtered out by three perceptrons with different object detection thresholds. The former perceptron uses a larger threshold for the initial screening, and this result is fed into the next perceptron with the smaller threshold; the final processing of the target in the perceptron with the smallest threshold is done to obtain the final detection box and classification, and a Fully Convolutional Network (FCN) [38] is concatenated to generate masks for semantic segmentation of the object.

**Figure 4:**
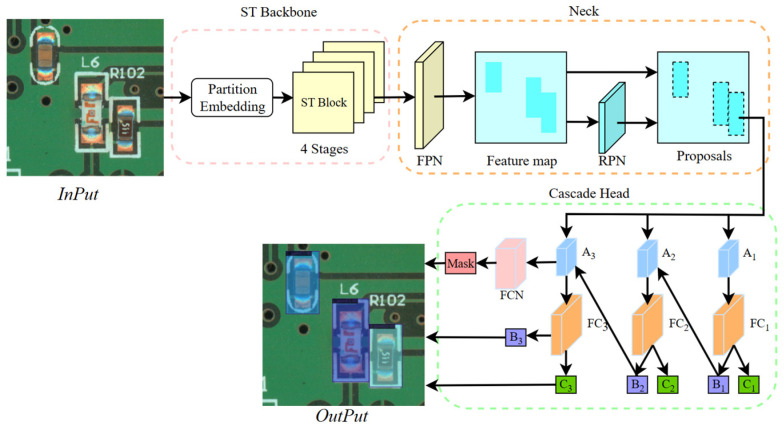
Defect detection model. The red dashed box shows the backbone structure of Swin Transformer; the orange dashed box shows the Neck structure using FPN for feature fusion; the green dashed box shows the Cascade Head architecture; A represents the ROI Align pooling layer at different stages, B is the detection box at different stages, C is the classification result at different stages.

Figure 4 illustrates our network model. At the input side of the network, the input PCB samples were resized to a fixed size using Patch Partition and partitioned into nonoverlapping patches, which were linearly encoded based on the positional information of the patches to generate Key matrices. Then, the low-dimensional feature information was fed into a two-layer Transformer Block for attention computation, and the weight of each patch with all other patches was computed in MSA, wherein the Value matrix was built.

Subsequently, feature extraction was performed based on the Query matrix and the thresholds obtained by training the network using the labeled dataset, and shallow feature information was computed by layer normalization.

Before sending the feature information to the next Transformer Block, a patch merging operation was performed to combine two adjacent patches, thus resulting in a higher-dimensional patch. This process generates high-dimensional information and creates a new Key matrix. An additional multihead self-attention (MSA) was used to compute the Value matrix for acquiring deeper feature information. The shallow network primarily identifies the finer features of the feature map, but dense patches are unable to capture global information, thus leading to significant computational redundancy. In contrast, the deep network merges neighboring patches, which reduces detection accuracy but allows for improved focus on global information. According to previous research [13], by counting the resolution of the data set image and the pixel ratio of the detected object in the picture, we chose the 4-stage architecture of the head network for feature extraction; too low a number of stages may not be able to fully mine the subtle feature information of the image, while too high a number of stages may result in the loss of the object’s contour information and at the same time greatly increase the number of parameters of the network. Compared to feature maps acquired through convolution and pooling, Transformers can extract information from images at various scales, thus balancing recognition accuracy and reducing computational complexity effectively. For feature information at different scales at each stage, these multiscale features were integrated using a Feature Pyramid Network (FPN) to generate a final feature map, which was then passed to a cascaded Mask R-CNN head for detection and segmentation.

The Cascade Head network has three different stages of perceptrons: the first stage uses a larger object detection threshold for initial screening of the detection boxes proposed by the RPN and undergoes a two-layer Fully Connected (FC) layer to obtain the initial classification result C_1_ and detection box B_1_. The results obtained in the first stage are used as inputs to the second stage perceptron to further optimize the results of the detection boxes by smaller thresholds and obtain B_2_ and C_2_, and B_2_ is input to the last perceptron for the final output detection box B_3_ and classification result C_3_. At the same time, a 4-layer fully convolutional network is concatenated to mask segment the final result and map it to the original image to get the final detection result.

## 4. Experimental Results

### 4.1. Automatic PCB Sample Generation Based on Stable Diffusion Model and ControlNet

This experiment was screened and expanded among 142 PCB samples collected from actual industrial production, of which 70 contain multiple detection objects and are nonindependent samples.

**Figure 5:**
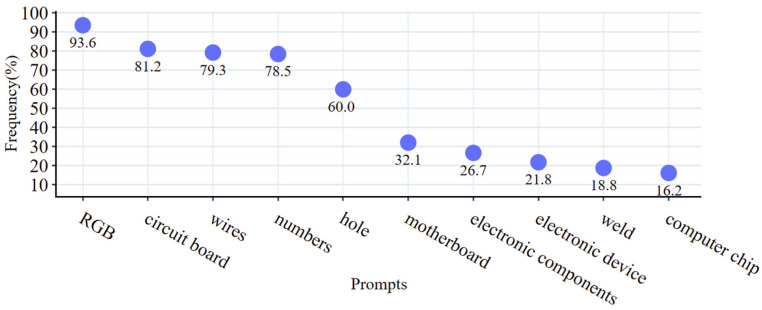
Frequency of occurrence of different prompts.

Firstly, we used CLIP to reverse derive the prompts for the samples containing the same objects and counted the frequency of occurrence of the prompts in all the samples. Figure 5 shows the ten prompts with the highest frequency of occurrence, and the most suitable prompts were selected as the auxiliary prompts for the subsequent Img2Img based on the experience and statistical data. After several attempts of comparison, we designed the final prompts as follows: a close up of a circuit board, motherboard, wires, many different numbers, hole, clear outline, weld, surface mount technology, solder paste, electronic components, electronic device, computer chip, red, blue, green, solder paste, automatically optical inspection, and the reverse prompts were selected as common prompt phrases.

**Figure 6:**
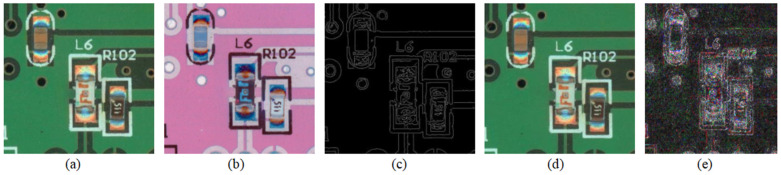
(**a**) Original input image; (**b**) corresponding inverse color map; (**c**) Canny feature map obtained using ControlNet; (**d**) result generated by Img2Img; (**e**) difference map obtained by subtracting (**a**) from (**d**).

We inverted the image (shown in Figure 6a) by subtracting the brightness value of each pixel using the maximum brightness in the image, thus making the bright parts darker and the dark parts brighter and changing the appearance and visual effect of the image. This enhanced the contrast of the image and made the details more prominent, thus making it easier to extract features when confronted with borders and solder joints against a green background. The inverse color processing of the image was achieved by transforming the pixel value matrix of the image as shown in Equation (6):

(6) $$M_{out}=L_{max}-M_{I}$$

where $M_{out}$ is the matrix of the pixel values of the output image, $L_{max}$ is the maximum value of luminance, and $M_{I}$ is the matrix of the pixel values of image *I*.

Edge feature extraction was performed on the input real samples and inverted color processed images (shown in Figure 6b) using ControlNet. Figure 6c shows the samples and feature extraction results. The original and extracted feature maps and corresponding prompts were input into the Stable Diffusion Model for automatic Img2Img sample generation, and sampling was performed using the DDIM in the open source v1-5-pruned-emaonly.ckpt model. The number of sampling steps was 20, the prompts guidance factor was set to seven, Control Net was enabled to control the output of the model, and the redraw intensity was 0.3. The generated samples are shown in Figure 6d, and Figure 6e demonstrates the disparity map obtained by subtracting the original image of the samples using the generated image (we increased the brightness of the disparity map to give it a better visual effect).

By comparing the generated samples with the real samples, we can see that there is a nonnegligible difference between the two at the pixel level, but they are visually indistinguishable. This shows that the generated sample images are in accordance with the objective facts, while the pixel level differences will have a new magnitude of influence on the calculation of the neural network after being converted into a tensor, which not only effectively avoids the occurrence of overfitting, but also improves the robustness of the method. Subsequently, the original image of the sample is labeled using the LabelMe labeling tool, and the label information of the original image has been combined with the generated sample image to obtain a brand-new set of training data, thus realizing the automatic expansion of the training sample.

When using this method to obtain samples, an arbitrary number of samples can be generated to meet the training requirements of the object detection network by changing the random seeds. In the detection of soldering defects on surface-mounted components on PCBs, we extracted the Canny features, Depth features, and HED features of the samples and used multiple sets of random seeds to expand the number of available sample datasets to 432, of which 180 contain multiple detection objects.

### 4.2. PCB Surface Mount Component Soldering Defect Detection

**Figure 7:**
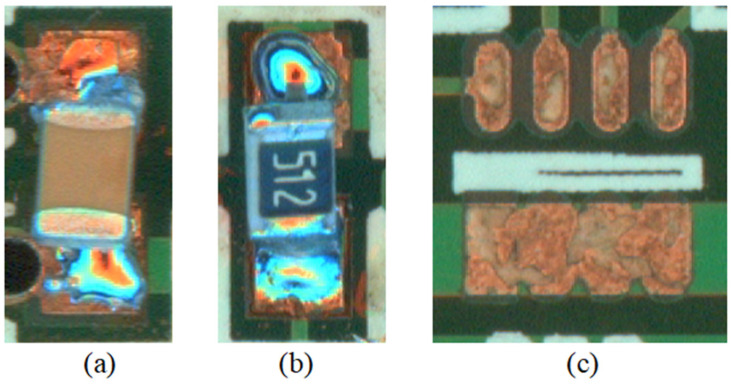
Some defective samples; (**a**) tombstone resistor; (**b**) shift capacitor; (**c**) missing potentiometer.

For the seven previously mentioned detection targets, we considered the missing, tombstone, and shift defects of each surface mount element according to the common defect types in actual industrial production (Figure 7 shows some of the defect samples), and we combined them with the normal situation to set a total of 18 categories for Label labeling. We found through preliminary experiments that the detection objects in the samples were 4w–8w pixels, and the pixel percentage was 6–12%, so when the thresholds of the three cascade-level perceptrons in Cascade Head were set to 0.5, 0.6, and 0.7 sequentially, it was able to maintain a small recall rate while achieving a high precision rate.

**Figure 8:**
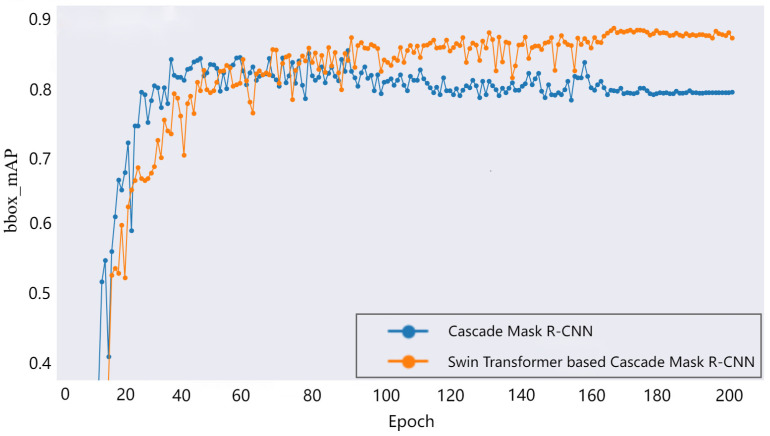
The bbox_mAP of Swin Transformer-based Cascade Mask R-CNN and original Cascade Mask R-CNN during training.

Using the 180 samples from the previous section and setting them in the ratio of 6:2:2 for the training, validation, and test sets, they were inputted into the Swin Transformer-based Cascade Mask R-CNN and ResNet-50-based Cascade Mask R-CNN for training, and the beach size was set to two, with a 0.0001 learning rate to train 200 epochs, while a training strategy of the learning rate warm-up and decay was employed using the AdamW [39] optimization algorithm for optimization. The model was implemented based on the Python 3.8.0 framework and run on a 12G NVIDIA 3080 (ASUS, Taiwan, China) graphics card for 1.5 h. Figure 8 shows the average localization accuracy of the detection box (bbox_mAP) obtained on the test set during the training of the two network models. After initial training, ResNet-50 was able to learn the feature information of the detection box quickly, thus showing its high degree of plasticity, but with deeper training, the Swin Transformer backbone was able to learn the multidimensional feature information of the object better, and new results for the Average Precision on different IoU scales were achieved.

After sufficient training, a comparison test was performed on the test set using the final two models. Comparing the mean Average Precision (mAP) and Average Recall (AR) of the detection boxes at different IoUs, our proposed network model improved the mAP of detection boxes by 0.016 (1.9%) over the original network at an intersection-parallel ratio. The class recognition accuracy improved by 0.041 (4.5%) at time and remained at the same level at time. The AR improved by 0.024 (2.8%). We trained and tested the same on the previous object detection network model, and Table 1 shows the test scores of the different models, where bbox_mAP is the Average Precision of the detection box at different IoU thresholds ranging from 0.50 to 0.95 (in steps of 0.05), and bbox_mAP_75 and bbox_mAP_50 are the category recognition rate mAPs of the detection box at IoU = 0.75 and IoU = 0.50, respectively.

**Figure 9:**
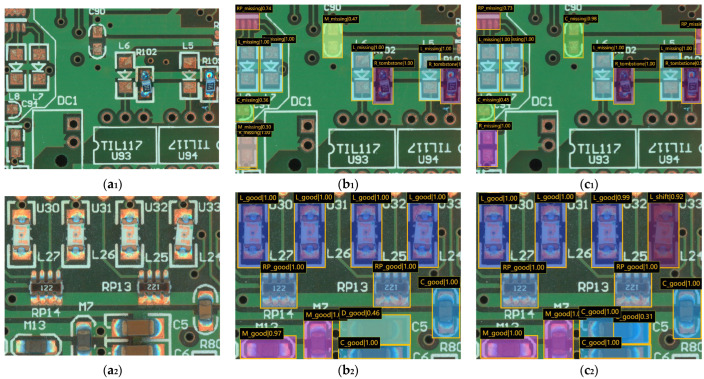

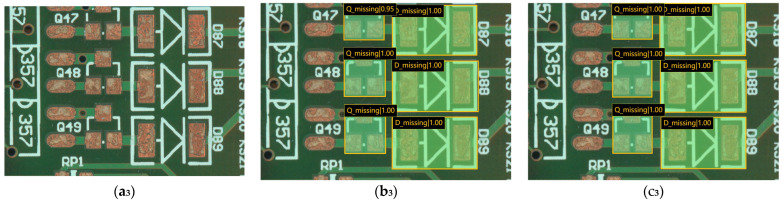
Comparison of detection results of different models, where (**a_1_**–**a_3_**) are the original images of the PCB samples; (**b_1_**–**b_3_**) are the test results using Cascade Mask R-CNN; (**c_1_**–**c_3_**) are the test results using our proposed method.

We show some of the detection results in Figure 9. Comparing the detection results of the two networks, it is intuitively found that the original Cascade Mask R-CNN with ResNet as the backbone was prone to misdetection when faced with multiple detection objects, e.g., misdetecting the missing capacitor as a missing thermistor and detecting the missing resistor as a missing thermistor at the same time in Figure 9(b_1_), as well as detecting the offset inductor as an inductor and the normal capacitor as a diode in Figure 9(b_2_), where it was detected as a normal inductor, and a normal capacitor was detected as a diode. In contrast, in our proposed model with Swin Transformer as the backbone, all the classes were successfully detected, and a high crossparallel ratio was maintained. The superiority of the Swin Transformer backbone to recognize subtle features has been verified.

## 5. Conclusions

Relying on the superior ability of the Stable Diffusion Model in the field of image generation and ControlNet’s logical control of large models, diffusion and the logical regeneration of complex images can be performed. The sample automatic generation method based on the Stable Diffusion Model and ControlNet proposed in this paper is capable of noncontrastive sample automatic generation for PCB samples with multiple objects. High-quality samples and datasets can be obtained quickly. The method can effectively address other target recognition areas that require large number of sample expansions and reduce the preparation cost of complex noncontrastive sample datasets.

At the same time, the Cascade Mask R-CNN network model proposed in this paper with Swin Transformer as the kernel can handle the complex multitarget detection tasks very well, relative to previous target detection algorithms, and obtain satisfactory detection accuracy for multiple objects at the same time. In the test results of the detection of soldering defects on surface mount components on the PCB, they showed that there was a performance that exceeded the previous Convolutional Neural Network model performance, thus improving the mean Average Precision by more than 1.9% and improving the Average Recall by 2.8%. This method can improve the defect detection capability in the field of multiobjective inspection, and the high accuracy of target pixel segmentation provides a reference for the optimization of subsequent production technology.

## Figures and Tables

**Table 1:** Results of different models on PCB surface mount component test set. (The best results achieved on this dataset are bolded).

Model	Params	mAP_bbox_	mAP_bbox__50	mAP_bbox__75	AR
Mask R-CNN [8]	45M	0.797	0.932	0.851	0.822
Cascade Mask R-CNN [9]	85M	0.840	0.912	0.912	0.857
ST-mask-rcnn [12]	50M	0.774	0.932	0.863	0.796
Our proposed method	91M	**0.856**	**0.953**	0.912	**0.881**

## Data Availability

The data presented in this study are available upon request from the corresponding author.
